# Targeted Mutagenesis in Plant Cells through Transformation of Sequence-Specific Nuclease mRNA

**DOI:** 10.1371/journal.pone.0154634

**Published:** 2016-05-13

**Authors:** Thomas J. Stoddard, Benjamin M. Clasen, Nicholas J. Baltes, Zachary L. Demorest, Daniel F. Voytas, Feng Zhang, Song Luo

**Affiliations:** Calyxt Inc., New Brighton, MN, United States of America; New England Biolabs, Inc., UNITED STATES

## Abstract

Plant genome engineering using sequence-specific nucleases (SSNs) promises to advance basic and applied plant research by enabling precise modification of endogenous genes. Whereas DNA is an effective means for delivering SSNs, DNA can integrate randomly into the plant genome, leading to unintentional gene inactivation. Further, prolonged expression of SSNs from DNA constructs can lead to the accumulation of off-target mutations. Here, we tested a new approach for SSN delivery to plant cells, namely transformation of messenger RNA (mRNA) encoding TAL effector nucleases (TALENs). mRNA delivery of a TALEN pair targeting the *Nicotiana benthamiana* ALS gene resulted in mutation frequencies of approximately 6% in comparison to DNA delivery, which resulted in mutation frequencies of 70.5%. mRNA delivery resulted in three-fold fewer insertions, and 76% were <10bp; in contrast, 88% of insertions generated through DNA delivery were >10bp. In an effort to increase mutation frequencies using mRNA, we fused several different 5’ and 3’ untranslated regions (UTRs) from *Arabidopsis thaliana* genes to the TALEN coding sequence. UTRs from an *A*. *thaliana* adenine nucleotide α hydrolases-like gene (At1G09740) enhanced mutation frequencies approximately two-fold, relative to a no-UTR control. These results indicate that mRNA can be used as a delivery vehicle for SSNs, and that manipulation of mRNA UTRs can influence efficiencies of genome editing.

## Introduction

In 2013, transgenic corn, soybean, and cotton varieties were planted on over 90% of the U.S. acreage [1]. These varieties were produced by stably integrating foreign DNA into the crop plants’ genome to confer novel phenotypes, such as herbicide tolerance or pest/pathogen resistance. Unfortunately transgenesis is not precise, and DNA integrates randomly into the genome, potentially leading to unintentional gene inactivation or variability in transgene expression. An alternative to traditional transgenesis is site-specific gene editing, wherein native plant genes are altered or inactivated to confer a trait of interest. The advent of sequence-specific nucleases (SSNs) has enabled plant genomes to be engineered with precision at high efficiency, thereby facilitating the development of non-transgenic plants with improved characteristics [2,3]. In general, the targeted DNA double-strand breaks created by SSNs are repaired by one of two mechanisms, achieving different types of genome edits: breaks repaired by non-homologous end joining (NHEJ) result in small insertions or deletions that are intended to knockout function of the target locus; breaks repaired by homologous recombination using a user-specified repair template result in precise edits to the genomic sequence of interest.

SSNs are typically delivered to plant cells as DNA constructs that either constitutively or transiently express the SSN. Common transformation methods, including protoplast transformation and biolistics, use large amounts of DNA to ensure high transformation efficiencies; however, this DNA can unintentionally integrate into the host genome. This is undesirable, since the goal of genome editing is typically to create a precise genome modification without off-target alterations, including unintended transgene integration. Further, prolonged expression of SSNs from DNA constructs can lead to off-target mutations. One method to decrease the likelihood of random DNA integration is to use purified SSN protein or protein/RNA complexes. Recent studies have shown that purified TALENs or Cas9/sgRNA can be directly transferred to plant cells to achieve targeted mutagenesis, thereby, circumventing the need to deliver DNA [4,5].

In animal systems, SSNs are frequently delivered as mRNA [6,7,8]; however, to the best of our knowledge, mRNA delivery of SSNs has not been tried in plant systems. For delivering SSNs to plant cells as mRNA, we reasoned it would be important to consider the choice of 5’ and 3’ UTRs, as they play a major role in translation efficiency [9], subcellular localization [10] and mRNA stability [11]. mRNA stability, in particular, can affect gene expression, as the half-life of the mRNA often dictates the amount of the corresponding protein that is produced. For example, the *cis*-acting elements in the 3′ UTR, such as AU-rich elements (AREs), are capable of modulating the stability and thereby the translation efficiency of mRNA transcripts [12,13].

Here, we assessed delivery of SSNs to plant cells using mRNA. We surveyed four different *A*. *thaliana* UTRs that were fused to the coding sequence of a highly-active TALEN pair. UTRs were chosen from mRNAs known to have long half-lives, and we specifically sought genes with diverse cellular functions [14]. Following PEG-mediated transformation of various mRNAs into *N*. *benthamiana* protoplasts, 454 pyrosequencing was used to assess mutagenesis at the TALEN target site. Targeted mutagenesis approximately 12-fold lower than DNA controls was observed, and certain UTRs increased the frequency of mutagenesis relative to the no-UTR control. Results from this study support the use of mRNA as a non-transgenic approach for genome editing, and they suggest that manipulation of UTRs can significantly influence the efficiency of genome editing.

## Materials and Methods

### Plasmid construction

**Fig 1:**
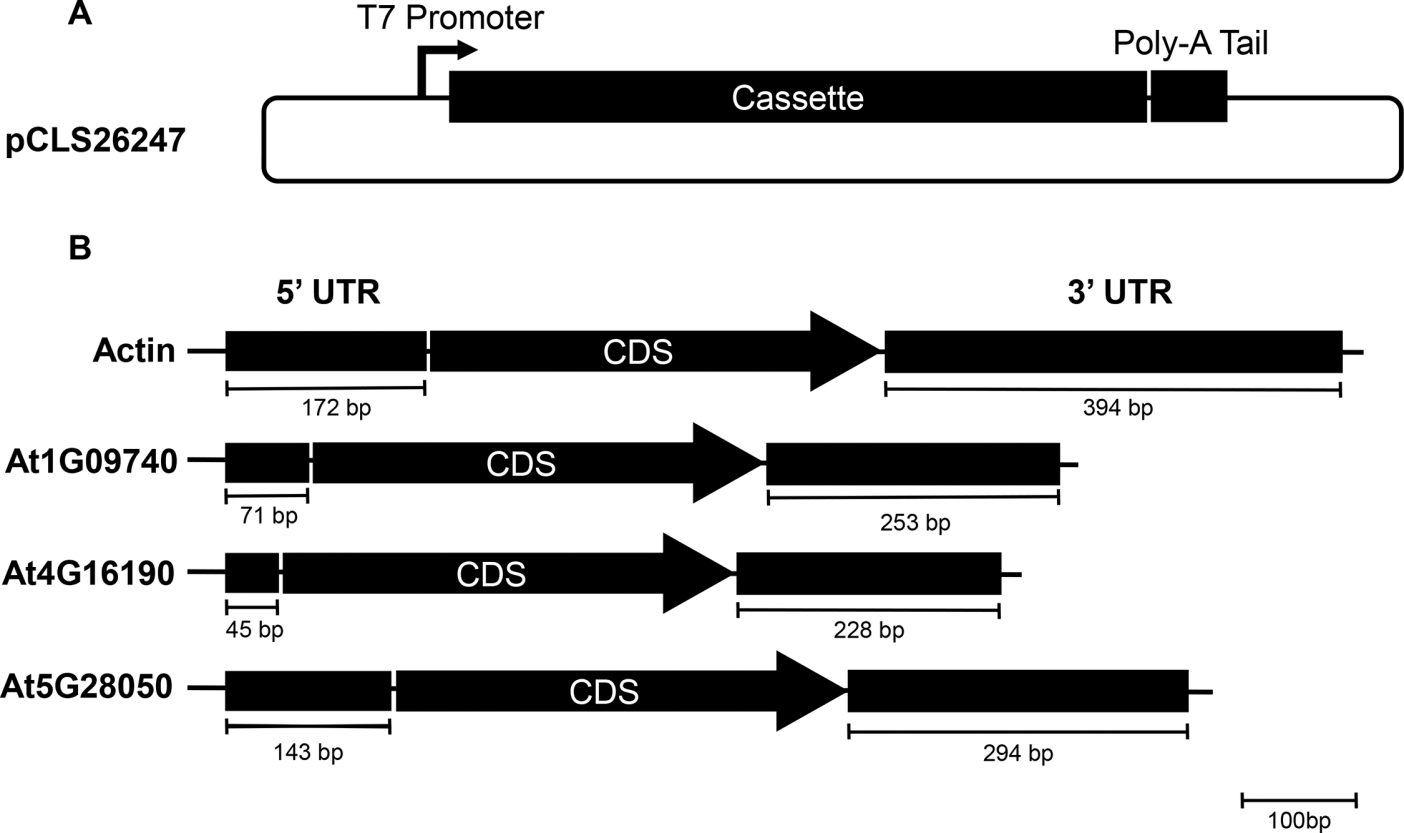
Schematic of mRNA expression constructs. A) The organization of a generic mRNA expression plasmid is shown with the T7 promoter, nuclease expression cassette and a poly-A sequence. B) Cassettes are illustrated that were used to express mRNA of the desired coding sequence (CDS). The sizes of the UTRs are given in bp. Note that the CDS is not drawn to scale.

A TALEN pair targeting the *ALS2* gene was previously described [4]. A plasmid was created to express TALEN mRNA by inserting a poly-A sequence into the mRNA expression plasmid pSP72 (Promega) by blunt-end ligation, giving rise to pCLS26247 (**Fig 1A)**. The 5’ UTRs were PCR-amplified and cloned into pCLS26247 using the restriction enzymes EcoRI and NcoI. The 3’ UTRs were amplified and cloned into pCLS26247 with the restriction enzymes XhoI and HindIII. Once the UTRs were successfully ligated into the backbone, the cassettes (YFP or TALEN subunits) were then cloned in between the UTRs using the restriction enzymes NcoI and XhoI (**Fig 1B**). The DNA expression plasmids were created using a plasmid backbone that contained a nopaline synthase (NOS) promoter and NOS terminator. The mRNA expression plasmids were used to amplify and clone the 5’ UTR, operably linked TALEN subunit, and the 3’ UTR into the DNA expression backbone plasmid using the restriction enzymes EcoRI and HindIII. All constructs were sequence-verified by Sanger sequencing.

### mRNA synthesis

The constructs containing the respective 5’ and 3’ UTRs and the no-UTR control were linearized by digestion with FspI, treated with proteinase K, and then purified using the DNA, RNA and protein purification kit (Macherey-Nagel). The eluted DNA was then quantified using a Nanodrop 2000c before *in vitro* mRNA synthesis by the mMESSAGE mMACHINE T7 Transcription Kit (Life Technologies). The mRNA transcripts were then purified using the RNeasy Mini Kit (QIAgen). After purification, the mRNA was quantified using the Quant-iT™ RiboGreen® RNA Assay Kit (Invitrogen™).

### Assessing TALEN activity in protoplasts

TALEN activity at endogenous target sites was measured in *N*. *benthamiana* protoplasts. Protoplast preparation was performed as described [15]. Nucleic acids were introduced into *N*. *benthamiana* protoplasts by polyethylene glycol- (PEG-) mediated transformation [16], including 1) plasmids containing TALEN coding sequences operably linked to the 5′ and 3′ UTRs (20 μg/monomer), or 2) mRNA transcripts encoding TALENs operably linked to the 5’ and 3’ UTRs (20 μg/monomer), or 3) a plasmid encoding YFP (20 μg), or 4) mRNA transcripts encoding YFP (20 μg). Twenty-four hours after treatment, transformation efficiency was measured using a fluorescent microscope to monitor YFP fluorescence in the sample transformed with the YFP plasmid. Two biological replicates were performed.

All samples of the transformed protoplasts were harvested two days post transformation, and genomic DNA was prepared using a hexadecyltrimethylammonium bromide- (CTAB-) based method. Using genomic DNA prepared from the protoplasts as a template, a 235-bp fragment encompassing the TALE-nuclease recognition site was amplified by PCR using primers (ALS2-F1 and ALS2-R1, seed sequence) and subjected to 454 pyrosequencing. Sequencing reads with insertion/deletion (indel) mutations in the spacer region were considered to be derived from imprecise repair of a cleaved TALEN recognition site by NHEJ. Mutagenesis frequency was calculated as the number of sequencing reads with NHEJ mutations out of the total sequencing reads. All data were analyzed using one-way ANOVA in Graphpad Prism v6.05. The mean of two replicates with different UTRs was compared to the mean of the no-UTR control population using the Dunnet's multiple comparison test with a significance level of alpha = 0.05.

### mRNA half-life assay

To measure mRNA half-lives, 200,000 protoplasts per sample were transformed with DNA expression vectors to compare the four different UTRs, no-UTR control, and positive 35S:YFP control. Fourteen hours post transformation, 100mg of actinomycin D, an antibiotic that can bind to DNA duplexes and inhibit RNA polymerase activity, was added to each sample. Time points were collected after the addition of actinomycin D at 0hr, 1hr, 2hr, 3hr and 4hr. Time points were immediately centrifuged at 1100rcf for five minutes before the supernatant was removed and the sample was flash-frozen in liquid nitrogen. To isolate the RNA the tissue was disrupted using a mortar and pestle while being kept chilled in liquid nitrogen. The total RNA was then purified by using the RNeasy Plant Mini Kit (QIAgen) followed by DNase treatment using DNase I (New England Biolabs® Inc). The half-life of the mRNA transcripts is estimated based on the first order half-life reaction ($t_{1/2}=\frac{ln2}{k}$), where k is the rate constant as determined by the slope of the ln [transcripts] as a function of time.

qRT-PCR was used to assess the TALEN mRNA levels at five different time points. Specific primers were designed to amplify a 99bp fragment of the FokI coding sequence within the TALEN constructs (FokI-L1 and FokI-R1). The PP2A gene (PP2A-L1 and PP2A-R1) was used as a housekeeping gene to normalize the TALEN mRNA copy number. qRT-PCR was performed on the purified RNA samples mentioned above using the SuperScript® III Platinum® One-Step qRT-PCR Kit (Invitrogen™) on the 7300 Real-Time PCR System (Applied Biosystems). qRT-PCR conditions per reaction were: 5 μM of each primer, 0.5uL of SuperScript®III RT, 12.5uL 2X SYBR® Green Reaction Mix, 0.5uL ROX Reference dye, 15ng of sample mRNA brought to a final volume of 25uL. The reactions were run using the Standard Cycling Program per the manufacturer’s recommendation. Primer efficiencies and Cq values were determined using the LingRegPCR v2013.0 software [17]. The results were normalized to the PP2A reference gene using an adapted version of the Microsoft Excel Qgene template [18]. Three technical replicates were performed for each sample.

## Results and Discussion

Transformation of plant cells with synthetic mRNA, as opposed to DNA, has potential to reduce random genetic modifications; however, there are limited reports of mRNA delivery in plants (outside of viral-based methods). To assess the efficacy of mRNA delivery, we sought to first deliver to protoplasts mRNA encoding yellow fluorescent protein (YFP). To this end, the YFP coding sequence was cloned into pCLS26247, a vector capable of generating mRNA by *in vitro* transcription (**Fig 1A**). The resulting construct was used to generate YFP mRNA, which served as the no-UTR control in the experiments described below.

We predicted that the addition of mRNA UTR sequences to YFP (or SSN) coding sequence would affect protein expression after delivery to plant cells. To test this hypothesis, four additional vectors were constructed, each with different 5’ and 3’ UTRs fused to the YFP coding sequence. The UTRs for all four UTR constructs were derived from *Arabidopsis thaliana* genes (**Fig 1B**; At1G09740, At4G16190, At5G28050 and actin 7; sequence information was obtained using the following GenBank accession IDs: NM_100846, AY136316, NM_001036882, and NM_121018, respectively). These genes were chosen because they express mRNAs with long half-lives. The half-lives of At1G09740, At4G16190, At5G28050 and actin 7 were previously determined to be 73.8h, 34.3h, 34.1h and 166.2h, respectively [14]. Further, we deliberately selected genes with diverse cellular functions, classified by FunCat [19], such as cellular structural organization, stress response, energy and metabolism. At1G09740 was specifically chosen because of its association with the ethylene stress response, which is common during the protoplast isolation and transformation process.

**Fig 2:**
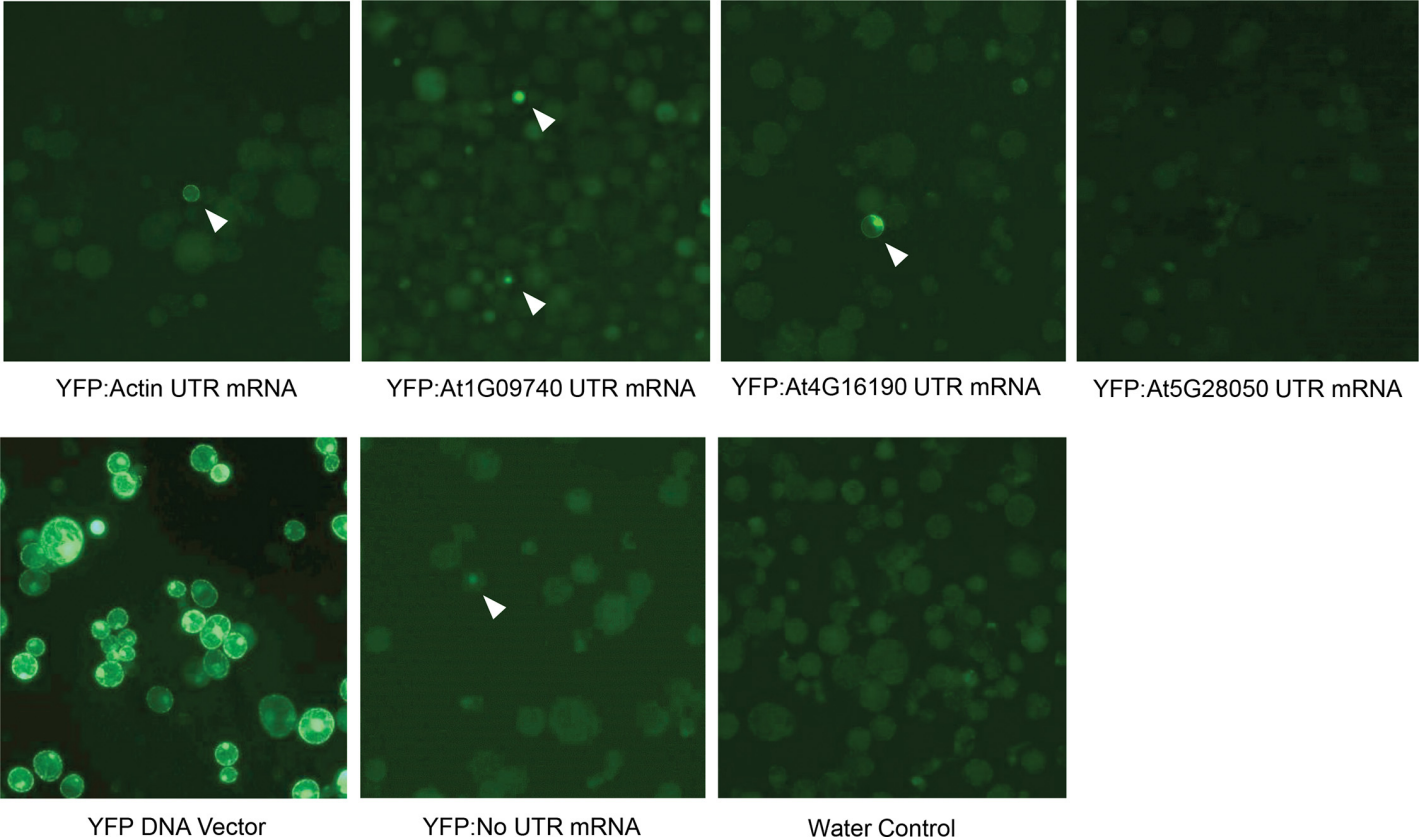
YFP expression in N. benthamiana protoplasts. Representative images of protoplasts ~24 hours after transformation. The top row shows protoplasts transformed with mRNA transcripts containing UTRs from the four genes tested. The lower row shows images for the controls, namely cells transformed with a DNA construct expressing YFP from a 35S promoter, YFP mRNA without UTRs and water. White arrowheads point to YFP expressing protoplasts.

Following *in vitro* transcription, 20 μg of YFP mRNA from the UTR and no-UTR vectors was introduced into *Nicotiana benthamiana* protoplasts using polyethylene glycol (PEG)-mediated transformation. Protoplasts were examined ~24 hours post transformation for YFP expression. YFP expression was observed in cells transformed with four of the five YFP mRNA constructs; we failed to detect YFP expression from the mRNA construct harboring At5G28050 UTRs. Further, relative to our plasmid DNA control, the total number of YFP-positive cells and the expression levels were low (**Fig 2**). We typically observed only ~1–5 YFP-positive cells among ~200,000 treated protoplasts, whereas our plasmid DNA control resulted in ~126,000 YFP-positive cells among ~200,000 (estimated based on an observed 63% transformation frequency). The number of YFP-positive cells from the transformation with YFP mRNA may not reflect the real number of transformed cells; that is, the threshold of YFP protein required for visualization may not have been reached, even though the cell was successfully transformed. Nonetheless, our results clearly demonstrate that delivery of mRNA to protoplasts and resulting protein expression can be achieved.

**Fig 3:**
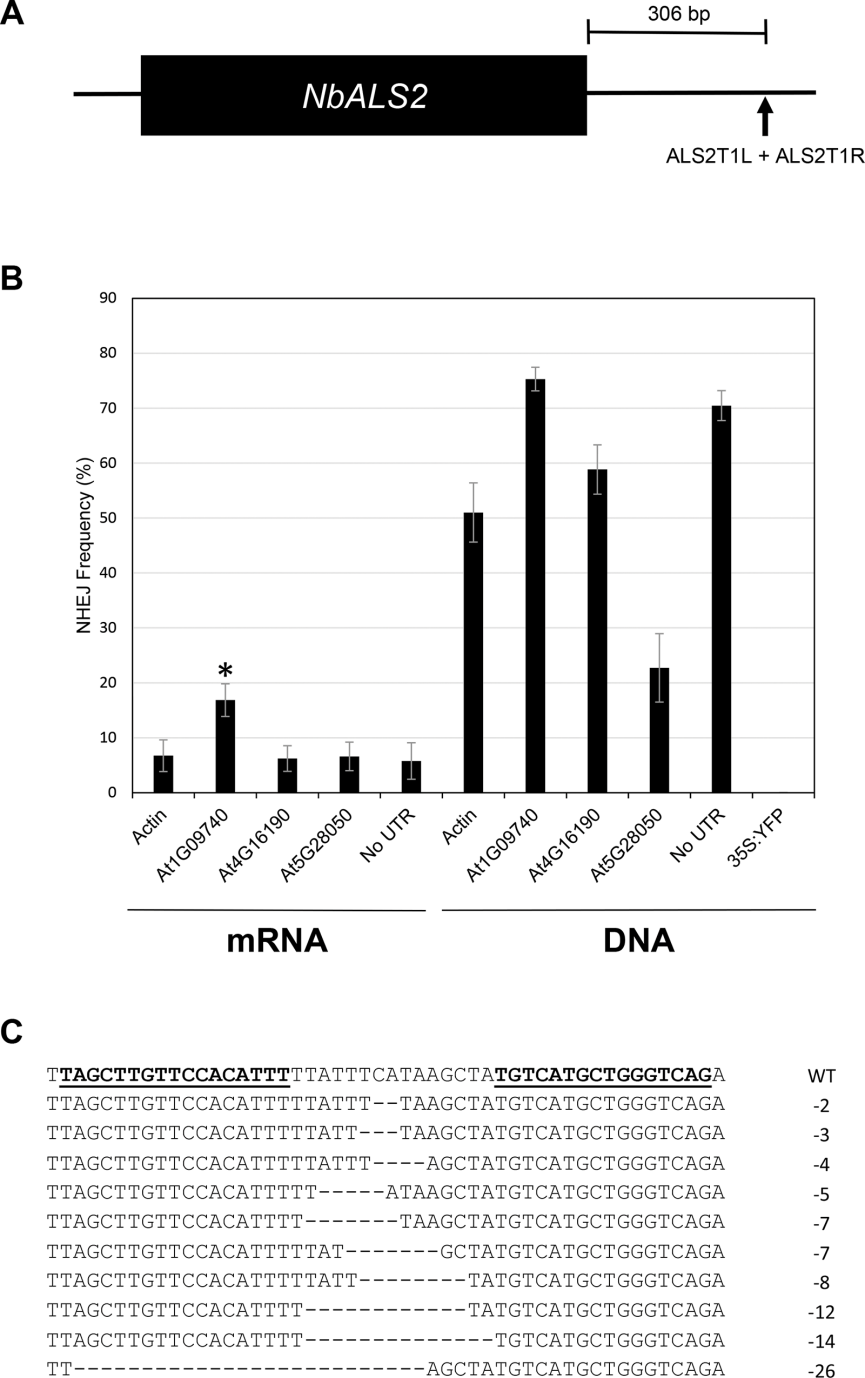
TALEN activity in *N*. *benthamiana* protoplasts. A) Schematic of *ALS2*, indicating the TALEN target site (black triangle). The target site is 306 bp downstream of the stop codon. B) Bar graph depicting the frequency (%) of NHEJ-induced mutations created by the ALS2T1 TALEN with the four different UTRs as well as the no-UTR control. Data for both mRNA and DNA constructs are presented. The asterisk denotes a sample that is significantly different from the no-UTR control (p = 0.0065). Error bars denote standard error. C) Representative NHEJ-induced mutations. In the wild-type (WT) sequence, the TALEN binding site is indicated as underlined, bold text. Representative mutations have the number of deleted bases indicated at the right.

To determine if mRNA can be used to deliver functional SSNs to plant cells, sequences encoding two TALEN monomers were cloned into each of the four UTR vectors for *in vitro* transcription and one no-UTR control vector. The TALEN pair recognizes a sequence downstream of the *acetolactate synthase 2 (ALS2)* gene of *Nicotiana benthamiana* (**Fig 3A**) [4]. Protoplasts were isolated and transformed with 20 μg of mRNA for each of the TALEN-monomers. As a control for mRNA delivery, we also transformed protoplasts with circular DNA constructs encoding the TALENs. The DNA constructs contained the same UTR sequences (or lack thereof) as the mRNA, and TALEN expression was driven by a NOS promoter. Genomic DNA was isolated from protoplasts ~48 hours post transformation, and the TALEN target site was amplified by PCR. The PCR product was then deep sequenced using 454 pyrosequencing. The transformation experiments were repeated to ensure reproducibility.

Sequenced amplicons were analyzed for insertions or deletions at the predicted TALEN target site. We observed evidence of TALEN-induced mutations in all samples transformed with DNA and mRNA; however, mutation frequencies were approximately 12-fold lower with mRNA (**Fig 3B**). The no-UTR DNA control resulted in 70.5% of all sequence reads with NHEJ-induced mutations. In contrast, the no-UTR mRNA control resulted in a 5.8% mutation frequency. Of the four *Arabidopsis* UTR pairs tested, the UTR pair from At1G09740 resulted in a significant (2.9-fold) increase in mutation frequency (16.9%) compared to the no-UTR mRNA control. Examples of mutations created with TALEN mRNA harboring the At1G09740 UTR pair are shown in **Fig 3C**. The remaining experimental samples had mutation frequencies comparable to the no-UTR controls. Together, these results demonstrate that SSNs can be delivered to plant cells as mRNA to achieve targeted mutagenesis, and that manipulation of the 5’ and 3’ UTR sequences can positively impact mutation frequencies.

One explanation for the observed differences in TALEN mutation frequencies is that the 5’ and 3’ UTRs confer differential mRNA stability, and subsequently, different levels of TALEN protein are produced in transformed protoplasts. We therefore predicted that TALEN mRNA harboring the At1G09740 5’ and 3’ UTR sequences (resulting in the highest levels of TALEN mutation frequencies) would have a longer half-life in protoplasts relative to the no-UTR mRNA control. To test this hypothesis, we performed a half-life assay with each of the UTR pairs in *Nicotiana benthamiana* protoplasts. However, we did not observe a significant correlation between half-life and mutation frequency (**S1 Fig**), suggesting that transcript stability is not responsible for the observed differences in mutation frequencies. Perhaps the UTRs contribute to translational efficiency.

**Fig 4:**
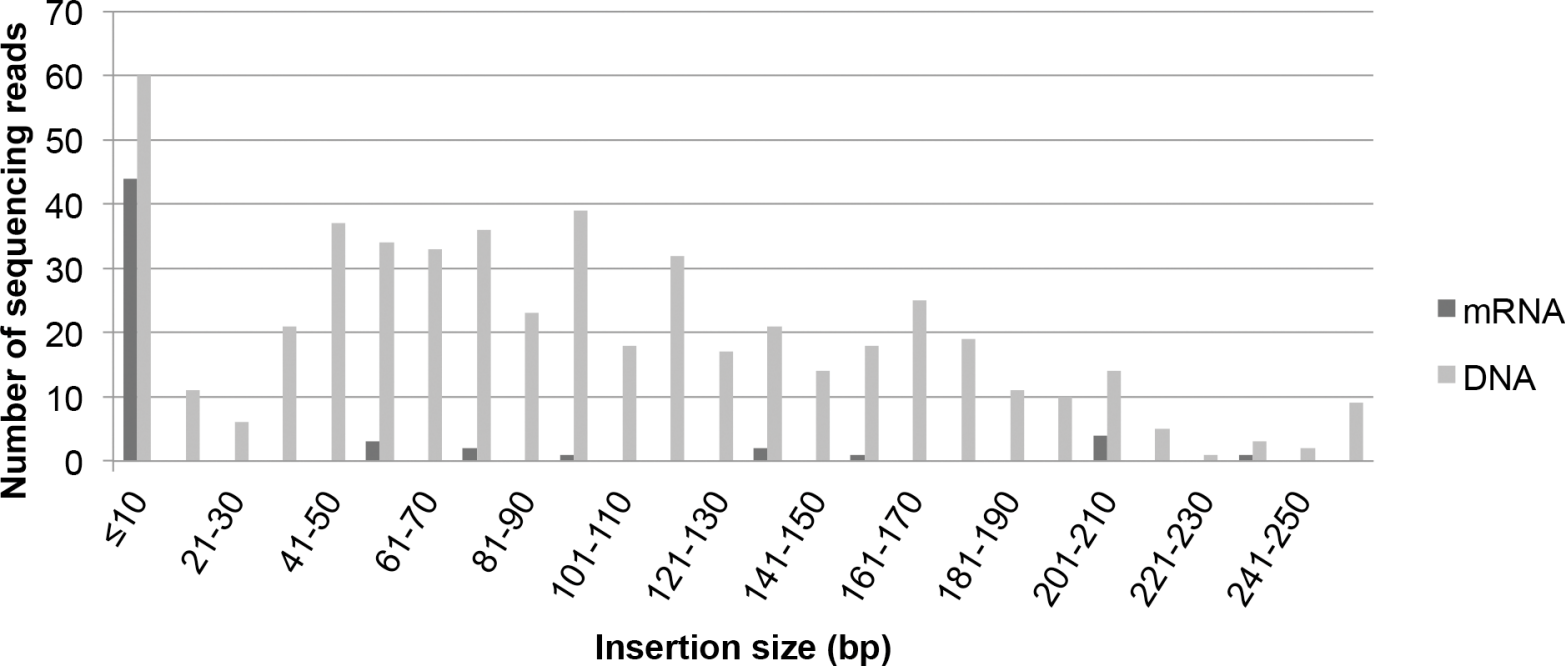
Mutation size and frequency for mRNA and DNA delivery of SSNs. The bar graph illustrates the unique insertion profiles between samples transformed with mRNA and DNA reagents.

As mentioned above, a benefit of using mRNA over DNA for nuclease delivery is that plants without foreign DNA are more likely to be created, and this may lessen the regulatory burden for crop varieties created through genome engineering. Consistent with this hypothesis, the analysis of the insertion/deletion (indel) mutation profile from the 454 pyrosequencing data revealed a large disparity in the types of mutations created by mRNA and DNA reagents. Cells transformed with DNA constructs had an average insertion frequency of 6.25% compared to a 1.98% insertion frequency for cells transformed with mRNA. Insertion frequencies were determined by dividing the number of sequences with insertions with the total number of reads containing mutations. Among the insertions created with DNA reagents, 88% were > 10bp with a median insertion size of 90 bp (**Fig 4**). This compares to mRNA delivery, where only 24% of insertions were > 10bp with a median insertion size of a mere 3 bp. For the DNA transformation experiments, the majority (>90%) of insertions ≥10 bp were sequences derived from the plasmid vector; that is, the inserted sequence originated from the TALEN-encoding plasmid. In contrast, only one of the insertions seen in the mRNA transformation experiments had an insertion (131bp) that matched the TALEN coding sequence. This insertion may have arisen from DNA contamination if the expression vector was not completely digested by DNase treatment after *in vitro* transcription. However, since the insertion matches the region of the TALEN construct that is expressed, it is also possible that TALEN mRNA acted as a template for DNA repair, as has previously been suggested for a spliced message that was incorporated at a SSN-induced break site in Drosophila [20]. Notably, potential DNA contamination is not expected to result in YFP or TALEN expression due to the inability of the T7 promoter to promote transcription in eukaryotic cells, including plant cells [21,22]. In summary, our data indicate that mRNA delivery yields mutation profiles that only rarely involve DNA insertion, and this is perhaps an advantage for creating plants with mutations and no foreign DNA.

## Conclusion

Here, we demonstrate that mRNA can be used as a reagent to deliver SSNs to plant cells to achieve targeted mutagenesis. Further, in contrast to DNA delivery, only a minority of the resulting mutations are insertions, and of these, only very rarely do they contain DNA sequence information captured from the SSN-encoding plasmid. Finally, the use of particular UTRs can improve the efficiency of SSNs-mediated mutagenesis, thereby providing an improved method of non-transgenic genome engineering.

## Supporting Information

S1 Fig mRNA half-life assay. Bar graph depicting the mRNA half-life time as determine via qRT-PCR. The y-axis is half-life in hours and the x-axis indicates the UTR that was fused to the TALEN subunits that were transformed into each sample.
(TIF)

S1 Table Primer information. This table gives the DNA sequence information for the primers used in this study. ALS2-F1 and ALS2-R1 are the seed sequences for the MID-tagged primers used for 454 pyrosequencing.
(XLS)

## References

[1] Fernandez-Cornejo J, Wechsler S, Livingston M, Mitchell L. Genetically Engineered Crops in the United States, ERR-162 U.S. Department of Agriculture, Economic Research Service, 2 2014.

[2] Jones HD. Regulatory uncertainty over genome editing. Nature Plants. 2015; 1: 1–3. 10.1038/nplants.2014.11 27246057

[3] Voytas DF, Gao C. Precision Genome Engineering and Agriculture: Opportunities and Regulatory Challenges. PLOS Biology. 2014; 12(6): 1–6. 10.1371/journal.pbio.1001877 PMC4051594 24915127

[4] Luo S, Li J, Stoddard TJ, Baltes NJ, Demorest ZL, Clasen BM, et al Non-transgenic Plant Genome Editing Using Purified Sequence-Specific Nucleases. Molecular Plant. 2015; 8:1425–1427. 10.1016/j.molp.2015.05.012 26074033

[5] Woo JW, Kim J, Kwon SI, Corvalán C, Cho SW, Kim H, et al DNA-free genome editing in plants with preassembled CRISPR-Cas9 ribonucleoproteins. Nature Biotechnology. 2015; 10.1038/nbt.3389 26479191

[6] Doyon Y, McCammon JM, Miller JC, Faraji F, Ngo C, Katibah GE, et al Heritable targeted gene disruption in zebrafish using designed zinc-finger nucleases. Nature Biotechnology. 2008; 26(6): 702–708. 10.1038/nbt1409 18500334 PMC2674762

[7] Meng X, Noyes MB, Zhu LJ, Lawson ND, Wolfe SA. Targeted gene inactivation in zebrafish using engineered zinc-finger nucleases. Nature Biotechnology. 2008; 26(6): 695–701. 10.1038/nbt1398 18500337 PMC2502069

[8] Sung YH, Baek I, Kim DH, Jeon J, Lee J, Lee K, et al Knockout mice created by TALEN-mediated gene targeting. Nature Biotechnology. 2013; 31(1): 23–24. 10.1038/nbt.2477 23302927

[9] Van der Velden AW, Thomas AAM. The role of the 5’ untranslated region of an mRNA in translation regulation during development. Int. J. Biochem. Cell Biol. 1999; 31:87–106. 10216946 10.1016/s1357-2725(98)00134-4

[10] Jansen RP. mRNA localization: message on the move. Nat. Rev. Mol. Cell Biol. 2001; 2:247–256. 11283722 10.1038/35067016

[11] Bashirullah A, Cooperstock RL, Lipshitz HD. Spatial and temporal control of RNA stability. Proc. Natl. Acad. Sci. 2001; 98(13):7025–7028. 11416182 10.1073/pnas.111145698 PMC34617

[12] Muhlrad D, Parker R. Mutations affecting stability and deadenylation of the yeast MFA2 transcript. Genes Dev. 1992; 6:2100–2111. 1427074 10.1101/gad.6.11.2100

[13] Brown CE, Sachs AB. Poly(A) Tail Length Control in *Saccharomyces cerevisiae* Occurs by Message-Specific Deadenylation. Mol. Cell. Biol. 1998; 18(11):6548–6559. 9774670 10.1128/mcb.18.11.6548 PMC109240

[14] Narsai R, Howell KA, Millar AH, O’Toole N, Small I, Whelan J. Genome-Wide Analysis of mRNA Decay Rates and Their Determinants in *Arabidopsis thaliana*. The Plant Cell. 2007; 19:3418–3436. 18024567 10.1105/tpc.107.055046 PMC2174890

[15] Wright DA, Townsend JA, Winfrey RJ, Irwin PA, Rajagopal J, Lonosky PM, et al High-frequency homologous recombination by zinc-finger nucleases. Plant J. 2005; 44:693–705. 16262717 10.1111/j.1365-313X.2005.02551.x

[16] Yoo SD, Cho YH, Sheen J. *Arabidopsis* mesophyll protoplasts: a versatile cell system for transient gene expression analysis. Nature Protocols. 2007; 2:1565–1572. 17585298 10.1038/nprot.2007.199

[17] Ruijter JM, Ramakers C, Hoogaars WM, Karlen Y, Bakker O, van den Hoff MJ, et al Amplification efficiency: linking baseline and bias in the analysis of quantitative PCR data. Nucleic Acids Res. 2009; 37:e45 10.1093/nar/gkp045 19237396 PMC2665230

[18] Muller PY, Janovjak H, Miserez AR, Dobbie Z. Processing of gene expression data generated by quantitative real-time RT-PCR. BioTechniques. 2002; 32:1372–1379. 12074169

[19] Ruepp A, Zollner A, Maier D, Albermann K, Hani J, Mokrejs M, et al The FunCat, a functional annotation scheme for systematic classification of proteins from whole genomes. Nucleic Acids Research. 2004; 32(18): 5539–5545. 15486203 10.1093/nar/gkh894 PMC524302

[20] Bozas A, Beumer KJ, Trautman JK, Carroll D. Genetic Analysis of Zinc-Finger Nuclease-Induced Gene Targeting in Drosophila. Genetics. 2009; 182: 641–651. 10.1534/genetics.109.101329 19380480 PMC2710147

[21] McBride KE, Schaaf DJ, Daley M, Stalker DM. Controlled expression of plastid transgenes in plants based on a nuclear DNA-encoded and plastid-targeted T7 RNA polymerase. Proceedings of the National Academy of Sciences. 1994; 91: 7301–7305. 10.1073/pnas.91.15.7301 PMC44387 8041784

[22] Fuerst TR, Niles EG, Studier FW, Moss B. Eukaryotic transient-expression system based on recombinant vaccinia virus that synthesizes bacteriophage T7 RNA polymerase. Proceedings of the National Academy of Sciences. 1986; 83: 8122–8126. 10.1073/pnas.83.21.8122 PMC386879 3095828

