# Supplementary figures and images for: Targeted Mutagenesis in Plant Cells through Transformation of Sequence-Specific Nuclease mRNA
Source: PLoS One. 2016 May 13;11(5):e0154634. doi: 10.1371/journal.pone.0154634 (PMC4866682; doi:10.1371/journal.pone.0154634)

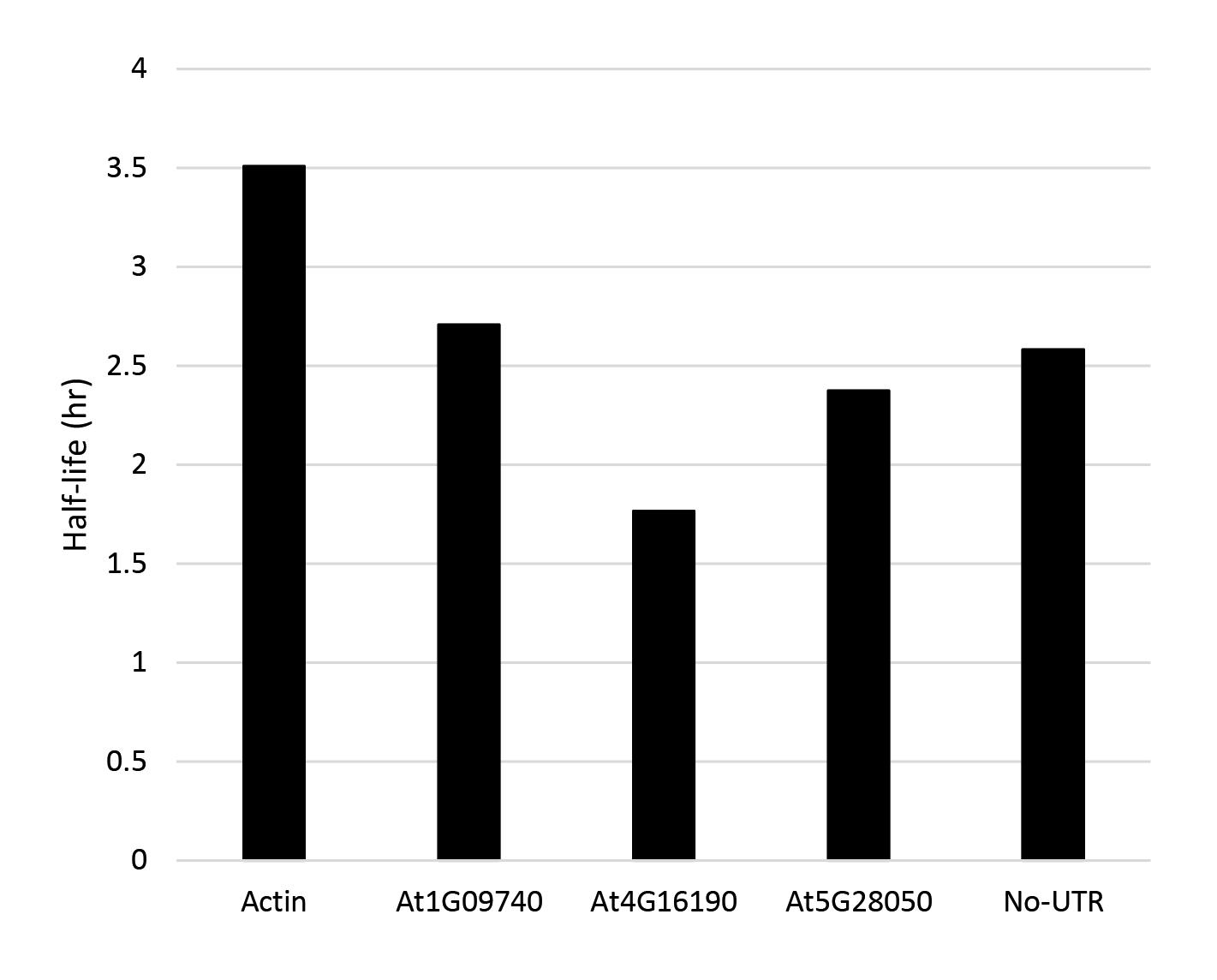

Supplement: S1 Fig — Bar graph depicting the mRNA half-life time as determine via qRT-PCR. The y-axis is half-life in hours and the x-axis indicates the UTR that was fused to the TALEN subunits that were transformed into each sample. (TIF) [file pone.0154634.s001.tif]
